# Comparison of white matter integrity between autism spectrum disorder subjects and typically developing individuals: a meta-analysis of diffusion tensor imaging tractography studies

**DOI:** 10.1186/2040-2392-4-25

**Published:** 2013-07-22

**Authors:** Yuta Aoki, Osamu Abe, Yasumasa Nippashi, Hidenori Yamasue

**Affiliations:** 1 Department of Neuropsychiatry, Graduate School of Medicine, The University of Tokyo, 7-3-1 Hongo, Bunkyo-ku, Tokyo 113-8655, Japan; 2 Department of Radiology, Nihon University School of Medicine, Itabashi-ku, Tokyo 173-8610, Japan; 3 Department of Radiology and Biomedical Engineering, Graduate School of Medicine, The University of Tokyo, 7-3-1 Hongo, Bunkyo-ku, Tokyo 113-8655, Japan; 4 Japan Science and Technology Agency, CREST, 5 Sanbancho, Chiyoda-ku, Tokyo 102-0075, Japan

**Keywords:** Autistic disorder, Asperger, Brain, Human, Imaging, Pervasive developmental disorder

## Abstract

**Background:**

Aberrant brain connectivity, especially with long-distance underconnectivity, has been recognized as a candidate pathophysiology of autism spectrum disorders. However, a number of diffusion tensor imaging studies investigating people with autism spectrum disorders have yielded inconsistent results.

**Methods:**

To test the long-distance underconnectivity hypothesis, we performed a systematic review and meta-analysis of diffusion tensor imaging studies in subjects with autism spectrum disorder. Diffusion tensor imaging studies comparing individuals with autism spectrum disorders with typically developing individuals were searched using MEDLINE, Web of Science and EMBASE from 1980 through 1 August 2012. Standardized mean differences were calculated as an effect size of the tracts.

**Results:**

A comprehensive literature search identified 25 relevant diffusion tensor imaging studies comparing autism spectrum disorders and typical development with regions-of-interest methods. Among these, 14 studies examining regions of interest with suprathreshold sample sizes were included in the meta-analysis. A random-effects model demonstrated significant fractional anisotropy reductions in the corpus callosum (*P* = 0.023, *n* = 387 (autism spectrum disorders/typically developing individuals: 208/179)), left uncinate fasciculus (*P* = 0.011, *n* = 242 (117/125)), and left superior longitudinal fasciculus (*P* = 0.016, *n* = 182 (96/86)), and significant increases of mean diffusivity in the corpus callosum (*P* = 0.006, *n* = 254 (129/125)) and superior longitudinal fasciculus bilaterally (*P* = 0.031 and 0.011, left and right, respectively, *n* = 109 (51/58)), in subjects with autism spectrum disorders compared with typically developing individuals with no significant publication bias.

**Conclusion:**

The current meta-analysis of diffusion tensor imaging studies in subjects with autism spectrum disorders emphasizes important roles of the superior longitudinal fasciculus, uncinate fasciculus, and corpus callosum in the pathophysiology of autism spectrum disorders and supports the long-distance underconnectivity hypothesis.

## Background

Atypical brain connectivity has repeatedly been implicated in neuroimaging studies of people with autism spectrum disorders (ASDs) (reviewed in [1]), and suggested as the source of atypical behavioral characteristics found in ASDs. Accumulated evidence from previous postmortem studies [2] and structural and functional magnetic resonance imaging (MRI) studies [3,4] provided the hypothesis that the autistic brain is characterized by long-distance underconnectivity [5-9]. However, it remains unclear which of the major seven long-distance tracts that compose the cortical network in the human brain is disordered [10-13]. These tracts include main association (intrahemispheric) fibers – namely the uncinate fasciculus (UF), cingulum, superior longitudinal fasciculus (SLF), inferior longitudinal fasciculus, inferior frontal occipital fasciculus (IFOF), and fornix – and one commissural (interhemispheric) fiber – namely the corpus callosum (CC).

Currently, diffusion tensor imaging (DTI) is one of the most powerful tools to investigate brain anatomical connectivity, and it can be used to study the orientation and integrity of white matter tracts (reviewed in [14]). This is accomplished by mainly estimating the degree of anisotropy, the property of being directionally dependent, the amount of water molecules using measures including fractional anisotropy (FA), the degree of anisotropy (ranges between 0 that means unrestricted in all directions, and 1 that means restricted in one direction), and the overall displacement of molecules using mean diffusivity (MD) [15].

There are two different principal approaches to evaluating anatomical connectivity from DTI data. The first approach uses a voxel-wise whole brain analysis (WBA), such as voxel-based analysis [16-18] or tract-based spatial statistics (TBSS) [19]. The other approach uses a region-of-interest (ROI) analysis, including using tractography to define the ROI [20-23]. WBA is a powerful strategy for detecting the location of white matter changes in any part of the brain. Although there has been debate regarding how WBA can verify that any given standard space voxel contains data from the same part of the same tract from each and every subject, it is expected that TBSS will resolve these problems [19]. Tractography is a relatively new technology that takes into account the orientation of the diffusion profile to reconstruct the trajectories of the whole fiber bundle and allows quantification of both white matter volume and microstructural organization in specific tracts [14].

Although DTI is a powerful means of testing the disconnectivity hypothesis of ASDs, a number of DTI studies of subjects with ASDs yielded inconsistent results. For example, although some studies reported FA reductions in the CC of subjects with ASDs [24-26], some reported no significant differences or even higher FA values [27,28] in the CC of subjects with ASDs compared with typically developing (TD) individuals. Further, previous studies have also yielded markedly inconsistent results regarding which tract shows robust differences between people with ASDs and TD individuals. Some studies reported FA differences in the CC of subjects with ASDs [24,27], whereas other reported differences in the inferior frontal occipital fasciculus or in the UF [24,28]. These inconsistencies in the direction and location of the reported changes in ASDs may at least partially result from an insufficient number of participants in each study.

To elucidate the tract that is disturbed in people with ASDs, we performed this systematic review and meta-analysis of DTI studies involving individuals with ASDs. As a number of previous neuroimaging studies have consistently reported structural and functional abnormalities in the frontal and temporal lobes (reviewed in [29,30]), we hypothesized that long-distance tracts and especially those with connections in the frontal and temporal lobes exhibit consistent abnormalities in people with ASDs. Based on this hypothesis, WBA studies were excluded from the current meta-analysis to specifically focus on the anatomical connectivity of a long-distance tract that is more amenable to evaluation by ROI analyses. The present study thus conducted a meta-analysis of the ROI-based DTI studies.

## Methods

### Systematic review

#### Data sources

DTI studies that examined the brains of individuals with ASDs and TD individuals were obtained from the computerized databases MEDLINE, Web of Science and EMBASE. The search terms used in the systematic screening were *autism, autistic*, *pervasive developmental disorder*, and *Asperger*, which were also combined with *diffusion*, *tensor*, *TBSS*, *tract-based spatial statistics* and *tractography*. Titles and abstracts of the studies were examined to verify whether they could be included. Reference lists of included articles were also examined to search for additional studies to be included.

#### Study selection

Studies were included in the initial database if they were brain DTI studies published between 1980 and August 2012 and they examined people with ASDs in comparison with a TD individuals. The studies were then included in the meta-analysis if they utilized a ROI method (for example, manually-defined ROI and tractography). The literature search was performed without language restriction. If the study did not report sufficient data, we emailed the corresponding author to obtain the data. In cases where this author did not respond, we excluded the study. Two reviewers (YA and YN) performed the study screenings independently.

#### Dividing studies into WBA and ROI studies

In the current review, we performed a systematic screening of DTI studies of subjects with ASDs. *A priori* defined localized approaches such as manually-placed ROI and tractography studies were then categorized as ROI studies, and were included in the current meta-analyses. However, as voxel-wise voxel-based analysis and TBSS studies are informative, these studies were categorized as WBA studies and are summarized in Table 1.

**Table 1:** Summary of studies included in the whole brain analysis diffusion tensor imaging group

					**Demographic character**												**Methodological character**											
**Study**	**ASD**				**TD**												**Value**						**Multiple comparison correction**					
	***N***^**a**^	**Males**	**Mean age (years)**	**IQ**^**b**^	***N***^**c**^	**Males**	**Mean age (years)**	**IQ**^**b**^	**Diagnostic tools**	**Medication / comorbidity**	**Tesla**	**B factor**	**Number of direction**	**NEX**	**TE/TR (ms)**	**Type of WBA**	**Software**	**FA**	**MD**	**RD**	**AD**	**ADC**	**Threshold**	**FWE**	**FDR**	**TFCE**	**Permutation**	**Main results**
**Whole brain voxel-wise analysis study**																												
Ameis and colleagues [43]	19	16	12.4	99	16	8	12.3	101	DSM-IV/ADI-R/ADOs	No/No	3	1,250	12	5	80/4,100	TBSS	FSL4.1	○	○	○	○	○	*P* <0.05	○	**×**	○	**×**	MD↓ in UF, IFOF, CC, SLF, in ASD children
Barnea-Goraly and colleagues [44]	7	7	14.6	101	9	9	13.4	107	ADI-R/ADOS	NA/Na	3	900	6	4	106/6,000	Voxel Based Whole Brain	SPM99	○	**×**	**×**	**×**	**×**	*P* <0.05	**×**	**×**	**×**	**×**	FA↓ in Cg, UF, in ASD
Barnea-Goraly and colleagues [18]	13	11	10.5	86	11	9	9.6	120	ADI-R/ADOS	NA/No	1.5	900	6	4	106/6,000	TBSS	FSL4.1	○	**×**	○	○	**×**	*P* <0.05	○	**×**	○	○	FA↓ in CC, SLF, in ASD
Bloemen and colleagues [45]	13	113	39	110	13	13	37	115	ICD-10/ADI-R/ADOS	NA/No	NA	1,300	4	NA	107/15,000	Voxel Based Whole Brain	SPM2	○	○	○	**×**	**×**	*P* <0.05	NA	NA	**×**	○	FA↓ in IFOF, ILF, CC, SLF, Cg, in ASD
Bode and colleagues [46]	27	20	14.7	NA	26	17	14.5	NA	ADI-R/ADOS	NA/No	NA	100	NA	1	90/8,000	TBSS	FSL	○	○	**×**	**×**	**×**	*P* <0.0005	NA	NA	**×**	**×**	FA↓ in IFOF
Cheung and colleagues [48]	13	12	9.3	100	14	13	9.9	112	ICD-10/ADI-R	No/No	1.5	1,200	25	NA	100/10,000	Voxel Based Whole Brain	SPM2	○	**×**	**×**	**×**	**×**	*P* <0.0005	**×**	**×**	**×**	**×**	FA↓ in SLF, FA, in ASD
Groen and colleagues [49]	17	14	14.4	98	25	22	15.5	105	ADI-R	No/No	1.5	900	30	4	93/10,100	Voxel Based Whole Brain	SPM5	○	○	**×**	**×**	**×**	*P* <0.05	**×**	○	**×**	**×**	FA↓ in SLF, ILF, in ASD
Jeong and colleagues [50]	32	29	439	NA	14	11	5.61	NA	DSM-IV	NA/No	3	1,000	6	6	NA/1,250	Whole Brain Tractography	FSL	○	○	**×**	**×**	**×**	Various	**×**	○	**×**	**×**	FA↓ in, AF <UF, CC in ASD
Jou and colleagues [51]	15	15	10.9	NA	8	8	11.5	NA	DSM-IV/ADI-ADOS	NA/NA	3	NA	30	3	85/6,200	TBSS	FSL	○	**×**	○	○	**×**	*P* <0.05	NA	NA	○	**×**	FA↓ in IFOF, SLF, UF, Cg, in ASD
Jou and colleagues [52]	10	10	13.5	91	10	10	13.5	105	DSM-IV/ADI-R/ADOS	NA/NA	1.5	1,000	6	6	92/1,1200	Voxel Based Whole Brain	Biolmage Suite	○	**×**	**×**	**×**	**×**	p<0.05	**×**	**×**	**×**	**×**	FA↓ in CC, CG, SLF, IFOF, ILF, in ASD
Ke and colleagues [53]	12	12	8375	101	12	12	9.4	100	DSM-IV/ADI-R	NA/No	1.5	1,000	15	2	104.4/8,000	Voxel Based Whole Brain	SPMS	○	**×**	**×**	**×**	**×**	p<0.05	**×**	**×**	**×**	**×**	No peak coordinates reported in cortical fibers
Kumar and colleagues [26]	32	29	5	NA	16	12	5.5	NA	DSM-IV	NA/No	3	1,000	6	6	79/1,0000	TBSS	FSL	○	**×**	**×**	**×**	○	p<0.05	**×**	**×**	**×**	**×**	FA↓ in rt UF, IFO, AF, rt Cg, CC in ASD
Noriuchi and colleagues [54]	7	6	14	93	7	6	13.4	116	DSM-IV	NA/NA	3	800	32	NA	88/7,420	Voxel Based Whole Brain	SPM2	○	**×**	**×**	**×**	**×**	p<0.05	○	**×**	**×**	**×**	FA↓ in SLF, CC, Cg, in ASD
Sahyoun and colleagues [55]	12	9	12.8	101	12	9	13.3	106	DSM-IV/ADI-R	No/NA	3	700	60	NA	84/7,980	TBSS	FSL	○	○	**×**	**×**	**×**	p<0.05	NA	NA	**×**	○	FA↑ in bl UF, rt SLF, FA↓ in bl, SLF, bl FOF in ASD
Thakkar and colleagues [56]	12	10	30	116	14	8	27	114	DSM-IV/ADI-R/ADOS	Yes/No	3	700	60	NA	82/8,400	Voxel Based Whole Brain	FSL	○	**×**	**×**	**×**	**×**	p<0.05	NA	NA	**×**	**×**	FA↓ in Cg in ASO
Weinstein and colleagues [27]	21	NA	3.3	NA	26	NA	3.3	NA	DSM-IV/ADI-R/ADOS	NA/No	1.5	1,000	15	2	94/1,1000	TBSS	FSL 0	○	○	○	○	**×**	p<0.05	NA	NA	○	**×**	FA↑ in lt SLF, bl Cg, CC in ASD

^a^Number of ASD participants. ^b^Mean IQ of participants. ^c^Number of controls. *AD* axial diffusivity, *ADC* apparent diffusion coefficient, *ADIR* Autism Diagnostic Interview-Revised, *ADOS* Autism Diagnostic Observation Schedule, *ASD* autism spectrum, *AF* arcuate fasciculus, *bl* bilateral, *CC* corpus callosum, *Cg* cingulum, *DSM-IV* Diagnostic and Statistical Manual of Mental Disorders-IV, *FA* fractional anisotropy, *FDR* false discovery rate, *FSL* FMRIB Software Library, *FWE* family wise error, *ICD-10* International Statistical Classification of Diseases and Related Health Problems-10, *IFOF* inferior frontal occipital fasciculus, *ILF* inferior frontal occipital fasciculus, *lt* left, *MD* mean diffusivity, *NA* not available, *NEX* number of excitation, *RD* radial diffusivity, *rt* right, *SLF* superior longitudinal fasciculus, *SPM* statistical parametric mapping, *TBSS* tract-based spatial statistics, *TD* typically developing, *TE* echo time, *TFCE* threshold, free cluster enhancement, *TR* repetition time, *UF* uncinate fasciculus, *WBA* whole brain analysis.

### Meta-analyses

#### Data extraction

To perform the meta-analyses, we defined the standardized mean differences as the effect size statistic Cohen’s *d*, which is calculated as the difference between the mean of the experimental group and that of the comparison group divided by the pooled standard deviation. In the current meta-analyses, the mean FA and MD in autistic individuals were subtracted from those in TD individuals in each ROI, and divided by the pooled standard deviation from both groups. Tracts that were reported in a minimum of three studies were included in the meta-analysis. We followed the guidelines for the Meta-analysis of Observational Studies in Epidemiology [31].

#### Analyses

All meta-analyses were performed using Comprehensive Meta-Analysis version 2 (Biostat, Inc., Englewood, NJ, USA). Cohen’s *d* was calculated and used as the effect size. In studies that used multiple ROIs in a single tract (for example, five ROIs in the CC [32]), the weighted average effect size was calculated to reflect the DTI value from the whole tracts rather than part of those tracts [33-35], and to consider differences among values from the same tract. Based on the results of previous studies that have reported asymmetry in major association fibers in subjects with ASD as well as in healthy subjects [36-38], we conducted meta-analyses for the right and left hemispheres independently. A random effects model was adapted for the meta-analysis to minimize potential heterogeneity from factors such as variations in the location of ROIs, magnetic field strength factors, and differences between ROI and tractography approaches. Studies that located their ROIs in the arcuate fasciculus were identified and integrated into analysis of the SLF, since the arcuate fasciculus is considered one of the subcomponents of the SLF [39]. The significance level was set at *P* <0.05.

#### Assessing between-study heterogeneity

The *I*^2^ statistics were calculated by the following formula:

$$I^{2}=\left(Q–\mathrm{df}\right)/Q\times 100$$

where *Q* is the chi-squared statistic and df its degrees of freedom [40]. The *I*^2^ statistics were employed to examine between-study heterogeneity. Based on previous studies, thresholds for the interpretation of *I*^2^, which can be misleading but a rough guide to interpretation, suggests that 0 to 50% represents mild heterogeneity, 50 to 75% moderate heterogeneity, and 75 to 100% considerable heterogeneity.

#### Sensitivity analyses

To determine the replicability and robustness of the findings, we performed one-study-removed sensitivity analyses in the meta-analyses that revealed significant differences. This analysis was adopted only when the number of included studies equaled or exceeded four, because fewer studies would preclude the conduct of a meta-analysis once one study was removed. In this approach, the meta-analysis is conducted through multiple permutations wherein one of the studies is removed from the dataset. This allows one to determine whether a single study is responsible for a significant result [41,42].

#### Publication bias

Publication bias was investigated qualitatively with a funnel plot for each group and brain region. Funnel plot asymmetry was assessed quantitatively by linear regression analysis. A significance level of *P* <0.10 was used to conclude that the studies were heterogeneous [40].

## Results

### Systematic review

#### Study selection

**Figure 1:**
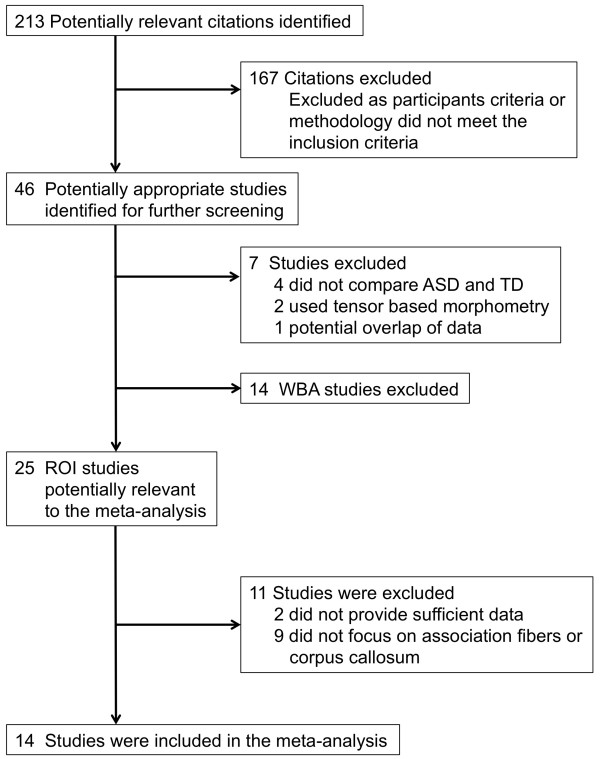
**The process used for the study selection.** ASD, autism spectrum disorder; ROI, region of interest; TD, typical development; WBA, whole brain analysis.

The literature search described above yielded 213 studies, of which 46 studies were identified as potential candidates for further screening. Four studies were excluded because they did not compare values between subjects with ASDs and TD individuals. Two studies were excluded because they were tensor-based morphometry studies. One study was removed because of potential overlap of the DTI data with another study from the same group. Seventeen studies were assigned into WBA DTI studies and are summarized in Table 1[18,26,27,43-56]. Three studies were assigned into both ROI and WBA studies [26,27,43]. Thus, 25 ROI DTI studies were identified as potentially relevant [24-28,32,43,57-74] (Figure 1 and Table 2).

**Table 2:** Summary of studies included in the regions-of-interest group

				**Demographic character**										**Methodological character**															
**Study**		**ASD group**		**TD group**													**Value**					**ROI tract**							
	***N***^**a**^	**Males**	**Mean age (years)**	**IQ**^**b**^	***N***^**c**^	**Males**	**Mean age (years)**	**IQ**^**b**^	**Diagnostic tools**	**Medication / comorbidity**	**Tesla**	**B factor**	**Number of direction**	**NEX**	**TE/TR (ms)**	**Type of WBA**	**FA**	**MD**	**RD**	**AD**	**ADC**	**CC**	**UF**	**Cg**	**SLF**	**ILF**	**IFOF**	**Fo**	**AF**
**Whole brain voxel-wise analysis study**																													
Alexander and colleagues [24]	43	43	16.2	107	34	34	16.4	113	DSM-IV/ICD-10/ADI-R/ADOS-G	Yes/Yes	3	1,000	12	4	84/7,000	ROI	○	○	○	○	**×**	○	**×**	**×**	**×**	**×**	**×**	**×**	**×**
Ameis and colleagues [43]	19	16	12.4	98.5	16	8	12.3	101	DSM-IV/ADI-R/ADOS	No/No	3	1,250	12	5	80/4,100	ROI	○	○	**×**	**×**	**×**	○	**×**	○	○	**×**	**×**	**×**	**×**
Beacher and colleagues [58]	28	15	32	NA	30	15	30	NA	DSM-IV	Yes/No	1.5	1,000	64	NA	95/8,000	ROI	○	**×**	**×**	**×**	**×**	○	**×**	**×**	**×**	**×**	**×**	**×**	**×**
Ben Bashat and colleagues [64]	17	NA	NA	NA	NA	NA	9.6	NA	DSM-IV/ADI-R/ADOS	NA/NA	1.5	6,000	6	4	128/1,800	ROI	○	○	**×**	**×**	**×**	○	**×**	○	○	**×**	**×**	**×**	**×**
Brito and colleagues [32]	8	8	9.53	NA	8	8	9.57	NA	DSM-IV	Yes/No	1.5	100	12	3	90/3,100		○	**×**	**×**	**×**	**×**	○	**×**	**×**	**×**	**×**	**×**	**×**	**×**
Catani and colleagues [67]	15	15	31	109	16	16	35	120	ICD-10/ADI/ADOS	NA/No	1.5	1,300	64	NA	107/1,500	Tractography	○	○	**×**	**×**	**×**	○	**×**	○	**×**	○	**×**	**×**	**×**
Cheon and colleagues [62]	17	17	11	112	17	17	10.2	114	DSM-IV/ ADI-R/ADOS	Yes/No	1.5	900	30	2	86/6500	ROI	○	○	**×**	**×**	**×**	**×**	**×**	**×**	**×**	**×**	**×**	**×**	**×**
Conturo and colleagues [74]	17	14	26.5	104	17	14	26.1	105	ADI-R/ADOS	Yes/No	1.5	NA	7	10	94/1,51750	Tractography	○	○	○	○	**×**	○	○	**×**	**×**	○	**×**	**×**	**×**
Fletcher and colleagues [59]	10	10	14.3	106	10	10	13.4	108	DSM-IV/ICD-10/ADI-R/ADOS	NA/NA	3	1,000	12	4	84/700	ROI	**×**	**×**	**×**	**×**	**×**	**×**	**×**	**×**	**×**	**×**	**×**	**×**	**×**
Hong and colleagues [60]	18	18	8.69	105	16	16	9.81	106	DSM-IV/ADI-R	No/No	1.5	1,000	15	2	104.4/8,000	ROI	○	**×**	**×**	**×**	○	○	**×**	**×**	**×**	**×**	**×**	**×**	**×**
Ingalhalikar and colleagues [69]	45	42	10.5	NA	30	14	10.3	NA	NA	NA/NA	3	1,000	15	NA	70/16,900	ROI	○	**×**	**×**	**×**	**×**	**×**	**×**	**×**	○	**×**	**×**	**×**	**×**
Knaus and colleagues [66]	14	14	16.1	103	20	20	14.1	116	DSM-IV/ADI-ADOS	NA/No	3	1,000	15	NA	N/A	Tractography	○	**×**	**×**	**×**	**×**	**×**	**×**	**×**	**×**	**×**	**×**	**×**	○
Kumar and colleagues [26]	32	29	5	NA	16	12	5.5	Na	DSM-IV	NA/No	3	1,000	6	6	79/10,000	Tractography	○	**×**	**×**	**×**	○	○	○	○	**×**	**×**	○	**×**	○
Langen and colleagues [73]	21	21	25.6	107	22	22	28.5	110	ICD-10/ADI-R/ADOS	No/No	3	1,300	32	NA	107/10,000	Tractography	○	○	○	○	**×**	**×**	**×**	**×**	**×**	**×**	**×**	**×**	**×**
Leemans and Jones [78]	43	43	16.2	108	34	34	16.4	113	DSM-IV/ICD-10/ADI-R/ADOS-G	NA/No	3	1,000	12	3	84/7,000	ROI	○	○	○	○	**×**	**×**	**×**	**×**	**×**	**×**	**×**	**×**	**×**
Lo and colleagues [25]	15	15	15.2	108	15	NA	15	111	DSM-IV/ICD-10/ADI-R	NA/No	3	6,000/4,000	102	NA	142/9,100, 130/9,600	ROI	○	**×**	**×**	**×**	**×**	○	○	○	**×**	**×**	**×**	**×**	○
Poustka and colleagues [63]	18	16	19.7	111	18	16	9.7	113	ICD-10/ADI/ADOS	NA/No	1.5	1,000	6	15	78/4,700	ROI	○	**×**	**×**	**×**	**×**	○	○	**×**	○	**×**	**×**	○	**×**
Pugliese and colleagues [57]	24	24	23.3	105	24	24	25.3	121	ICD-10/ADI-R	No/No	NA	1,300	32	NA	107/15,000	Tractography	○	○	**×**	**×**	**×**	**×**	○	○	**×**	○	○	○	**×**
Sahyoun and colleagues [71]	9	7	12.8	101	12	9	13.3	106	ADIM-IV/ADI-R	No/No	3	700	60	Na	84/7,980	ROI	○	**×**	**×**	**×**	**×**	**×**	**×**	**×**	**×**	**×**	**×**	**×**	**×**
Shukla and colleagues [65]	24	24	5	NA	16	12	5.9	NA	ADI-R/ADOS	Yes/No	3	200	15	4	99.4/1,0000	ROI		○	○	○	**×**	○	**×**	**×**	**×**	**×**	**×**	**×**	**×**
Sivaswamy and colleagues [68]	27	24	5	NA	16	12	5.9	NA	DSM-IV	Yes/No	3	1,000	6	6	160/11000	ROI	○	**×**	**×**	**×**	**×**	**×**	**×**	**×**	**×**	**×**	**×**	**×**	**×**
Sundaram and colleagues [72]	50	43	4.79	NA	16	11	6.84	NA	DSM-IV0	Yes/No	3	1,000	6	6	79/10,000	Tractography	○	**×**	**×**	**×**	○	**×**	**×**	**×**	**×**	**×**	**×**	**×**	**×**
Thomas and colleagues [28]	12	12	28.5	107	18	18	22.4	112	DSM-IV/ ADI-R/ADOS	NA/No	3	850	6	12	82/4,900	ROI	○	**×**	**×**	**×**	**×**	**×**	○	**×**	**×**	○	○	**×**	**×**
Verhoeven and colleagues [61]	19	16	13.8	NA	33	24	12.9	NA	DSM-IV	Yes/No	3	800	45	2	55/11,043	Tractography	○	**×**	**×**	**×**	○	**×**	**×**	**×**	○	**×**	**×**	**×**	**×**
Weinstein and colleagues [27]	22	NA	3.2	NA	28	NA	3.6	NA	DSM-IV ADI-R/ADOS	NA/Yes	1.5	1,000	15	2	94/11,043	Tractography	○	○	○	○	**×**	○	**×**	○	○	**×**	**×**	**×**	**×**

^a^Number of ASD participants. ^b^Mean IQ of participants. ^c^Number of controls. *AD* axial diffusivity, *ADC* apparent diffusion coefficient, *ADIR* Autism Diagnostic Interview-Revised, *ADOS* Autism Diagnostic Observation Schedule, *AF* arcuate fasciculus, *ASD* autism spectrum disorder, *CC* corpus callosum, *Cg* cingulum, *DSM-IV* Diagnostic and Statistical Manual of Mental Disorders-IV, *FA* fractional anisotropy, *Fo* fornix, *ICD-10* International Statistical Classification of Diseases and Related Health Problems-10, *IFOF* inferior frontal occipital fasciculus, *ILF* inferior longitudinal fasciculus, *MD* mean diffusivity, *NA* not available, *NEX* number of excitation, *RD* radial diffusivity, *ROI* region of interest, *SLF* superior longitudinal fasciculus, *TD* typically developing, *TE* echo time, *TR* repetition time, *UF* uncinate fasculus.

#### Characteristics of WBA DTI studies

Seventeen WBA-DTI studies involved 287 individuals with ASDs and 258 TD individuals. Eight studies utilized a 3 T scanner [26,43,44,50,51,54-56], whereas seven studies used a 1.5 T scanner [18,27,47-49,52]. Two studies did not disclose the strength of the magnetic field [45,46]. Eight studies adopted TBSS [18,26,27,43,46,47,51,55]. Thirteen studies identified coordinates where FA was significantly reduced in the CC or association fibers in individuals with ASDs [18,26,44,45,47-52,54-56], whereas five studies showed coordinates where FA was significantly increased in the CC or association fibers in individuals with ASDs [27,46-48,55].

### Meta-analyses

#### Selection of studies

Among the 25 candidate ROI DTI studies, two studies were excluded because they did not provide mean and standard deviation metabolite levels; instead of reporting these levels, one reported only *P* values [64,65] while the other reported only the correlation to autism quotient [66]. These studies were excluded after we emailed the corresponding author to obtain data to calculate the effect size, and the authors did not respond or provide them. In addition, nine studies were excluded because they did not focus on association fibers; instead, they focused on cerebellar fibers [67,68], short distance fibers [69-72], projection fibers [73], and the amygdalo-fusiform and hippocampo-fusiform pathways [74]. Following these exclusions, 14 studies that examined 330 subjects with ASDs and 313 TD individuals were included in the current meta-analysis [24-28,32,43,57-63].

#### Meta-analyses

##### Corpus callosum

**Figure 2:**
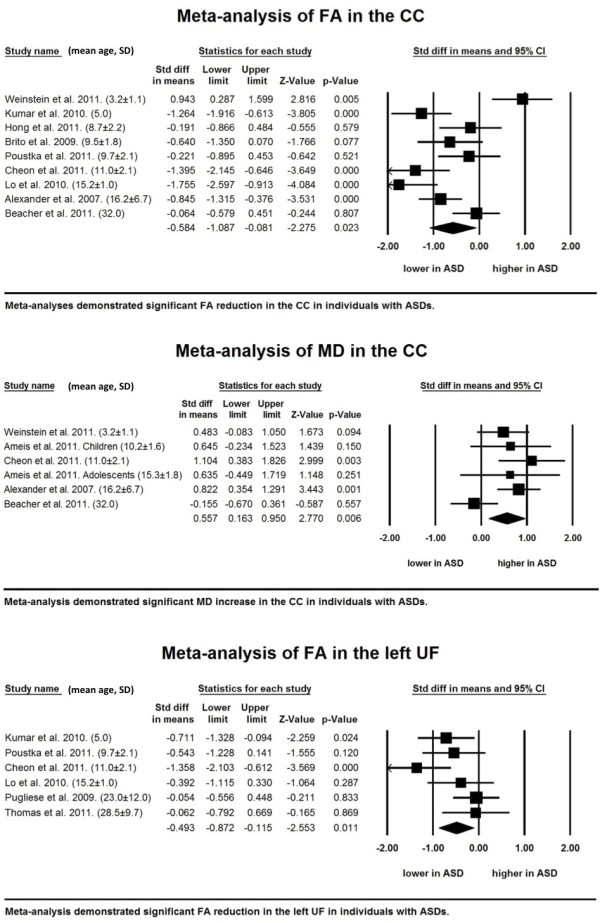
**Significant findings from the meta-analyses in the corpus callosum and left uncinate fasciculus.** Forest plots of the meta-analyses of fractional anisotropy (FA) (upper), mean diffusivity (MD) in the corpus callosum (CC) (middle) and MD in the left uncinate fasciculus (UF) (lower). Mean and standard deviation (SD) of age of individuals with autism spectrum disorders (ASDs) are demonstrated at the end of each study name. Studies are lined in the order of mean age from the youngest (top) to the oldest (bottom).

Nine studies that recruited 208 subjects with ASDs and 179 TD independent individuals were integrated into the meta-analysis of FA values in the CC [24-27,32,58,60,62,63]. We found significantly low FA values in subjects with ASDs compared with TD individuals (*P* = 0.023). Although the meta-analysis showed considerable heterogeneity, there was no publication bias. For the MD data, five studies with six datasets that examined 129 subjects with ASDs and 125 TD individuals were included in the meta-analysis, and this analysis found significantly increased MD values in subjects with ASDs (*P* = 0.006) [24,27,43,58,62]. (Figure 2 and Table 3; see Additional file 1).

**Table 3:** Meta-analysis by tract and value

**Tract**	**Laterality**	**Value**	**Number of dataset**	***N***^**a**^	***N***^**b**^	**Lower 95% CI**	**Upper 95% CI**	***Z*value**	***P*value**	**Heterogeneity*I***^**2**^	**Publication bias**
Corpus callosum	NA	FA	9	208	179	−1.087	−0.081	−2.275	0.023	81.91	0.541
		MD	6	129	125	0.163	0.950	2.770	0.006	53.81	0.650
Urinate fasciculus	Left	FA	6	117	125	−0.872	−0.115	−2.553	0.011	49.76	0.279
		MD	4	60	75	−0.148	1.300	1.584	0.113	73.96	0.682
	Right	FA	6	116	124	−0.998	0.203	−1.237	0.216	75.81	0.027
		MD	4	60	75	0.123	−0.410	0.963	0.790	69.11	0.636
Cingulum	Left	FA	5	101	109	−0.846	0.224	−1.139	0.255	69.80	0.633
	Right	FA	5	101	109	−00.475	0.525	0.099	0.921	65.94	0.857
Superior longitudinal fasciculus	Left	FA	5	96	86	−0.954	−0.097	−2.404	0.016	48.34	0.707
		MD	4	51	58	0.062	1.256	2.163	0.031	49.66	0.950
	Right	FA	5	92	82	−0.682	0.158	−1.229	0.219	43.99	0.462
		MD	4	51	58	0.140	1.100	2.534	0.011	26.11	0.499
Inferior longitudinal fasciculus	Left	FA	4	61	85	−1.491	0.313	−1.279	0.201	83.19	0.907
	Right	FA	4	61	85	−0.775	0.000	−1.959	0.050	20.15	0.207
Inferior frontal occipital fasciculus	Left	FA	3	68	76	−0.979	0.195	−1.309	0.191	63.22	0.690
	Right	FA	3	68	76	−0.735	0.152	−1.287	0.198	37.41	0.528

^a^Number of ASD participants. ^b^Number of controls. *ASD* autism spectrum disorder, *CI* confidence interval, *FA* fractional anisotropy, *MD* mean diffusivity.

##### Uncinate fasciculus

Six studies with six datasets were included in the meta-analysis of 117 subjects with ASDs and 125 TD individuals [25,26,28,57,62,63]. The random-effects model demonstrated a significant FA reduction in the left hemisphere in people with ASDs (*P* = 0.011) (Table 3). The between-study heterogeneity was low and there was no publication bias (see Additional file 1). The analysis demonstrated no significant differences in FA in the right hemisphere between subjects with ASDs and TD individuals (*P* = 0.216). For the MD data, our integrated analysis of three studies with four datasets with 60 subjects with ASDs and 75 TD individuals revealed no significant difference between subjects with ASDs and TD individuals in either hemisphere (*P* = 0.113 and 0.790, left and right, respectively) [43,57,62].

##### Cingulum

Datasets from five studies (involving 101 subjects with ASDs and 109 TD individuals) investigating FA values in the cingulum were included in the analysis [25-27,32,57]. A meta-analysis of these studies showed no significant differences between subjects with ASDs and TD individuals in the left (*P* = 0.255) and right (*P* = 0.921) hemispheres (Table 3; see Additional file 1). Only two studies reported MD in the cingulum in the left and right hemispheres, separately [27,57]. We therefore did not conduct a meta-analysis for MD in the cingulum.

##### Superior longitudinal fasciculus

**Figure 3:**
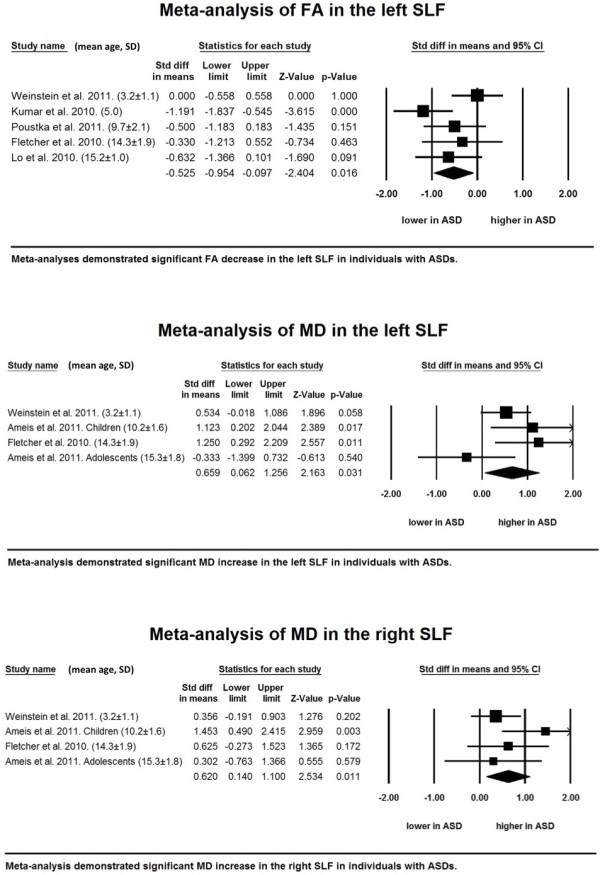
**Significant findings from the meta-analyses in the superior longitudinal fasciculus.** Forest plots of the meta-analyses of fractional anisotropy (FA) (upper) and mean diffusivity (MD) (middle) in the left superior longitudinal fasciculus (SLF) and MD in the right SLF (lower). Mean and standard deviation (SD) of age of individuals with autism spectrum disorders (ASDs) are demonstrated at the end of each study name. Studies are lined in the order of mean age from the youngest (top) to the oldest (bottom).

Five studies that recruited 96 subjects with ASDs and 86 TD individuals were examined in the meta-analysis for FA [25-27,59,63]. The random-effects model revealed a significant decrease in FA values in the left hemisphere (*P* = 0.016). By contrast, a meta-analysis of findings for the right hemisphere demonstrated no significant difference (*P* = 0.219). Three studies with four datasets that involved 51 individuals with ASDs and 58 TD individuals were integrated into the meta-analysis for MD [27,43,59]. The results revealed a significant MD increase in the left (*P* = 0.031) and right (*P* = 0.011) hemispheres without publication bias and minimal between-study heterogeneity (Table 3 and Figure 3; see Additional file 1).

##### Inferior longitudinal fasciculus

A meta-analysis of four studies with four datasets with 61 subjects with ASDs and 85 TD individuals did not demonstrate significant FA reductions in the ASD group in the left (*P* = 0.201) and right (*P* = 0.050) hemispheres [28,32,57,62]. A meta-analysis for MD values in this tract could not be conducted because of the limited number of studies. The meta-analysis of FA values demonstrated moderate heterogeneity but no publication bias (Table 3; see Additional file 1).

##### Inferior frontal occipital fasciculus

Three studies with three datasets that involved 68 subjects with ASDs and 76 TD individuals were examined in the meta-analysis [26,28,57]. The meta-analysis did not show any significant FA reductions in people with ASDs in either hemisphere (*P* = 0.191 and 0.198, left and right, respectively) (Table 3; see Additional file 1). MD data were available from only one study.

#### Sensitivity analyses

To test the robustness of the significance of findings, we conducted one-study-removed sensitivity analyses of FA values in the CC, left UF and left SLF, and MD values in the CC, left UF, left and right SLF that included more than four studies. For FA values in the CC, five sensitivity analyses out of nine preserved the significant FA reductions, and the remaining four analyses preserved reductions that approached significance (see Additional file 2). One-study-removed sensitivity analysis of FA values in the left UF demonstrated that five sensitivity analyses out of six reached significance whereas the remaining one did not (see Additional file 2). All of the one-dataset-removed sensitivity analyses of MD values in the CC preserved the significance of the higher MD values in subjects with ASDs (Additional file 2). With regard to the left SLF, two out of five one-study-removed sensitivity analyses demonstrated a significant FA reduction. Only one out of four one-study-removed sensitivity analyses preserved the significance of the MD increase in the left SLF. Two out of four one-study-removed sensitivity analyses demonstrated a significant MD increase in the right SLF (see Additional file 3).

## Discussion

**Figure 4:**
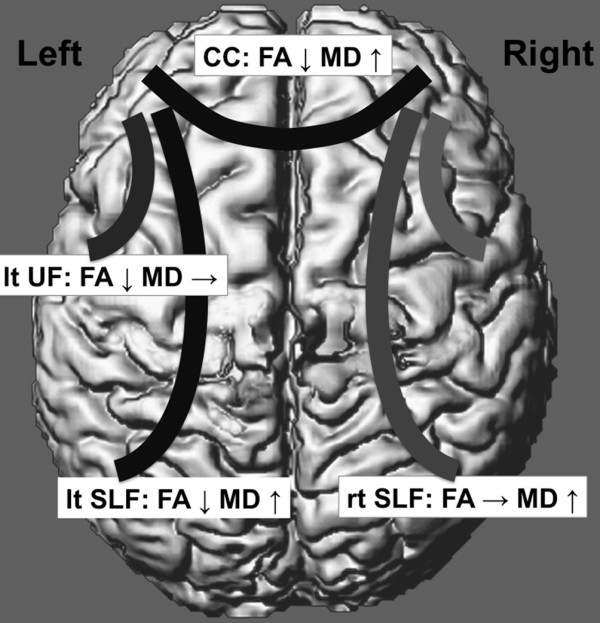
**Summary of the main findings.** The fractional anisotropy (FA) was significantly decreased, whereas the mean diffusivity (MD) was significantly increased in the corpus callosum (CC) in subjects with autism spectrum disorders (ASDs) compared with TD individuals. The FA was significantly decreased in individuals with ASDs in the left (lt) but not in the right (rt) uncinate fasciculus (UF). There was no significant difference in MD values in the UF between subjects with ASDs and TD individuals. The FA was significantly decreased in the left superior longitudinal fasciculus (SLF) but not in the right SLF. The MD was significantly increased in the SLF bilaterally.

To our knowledge, this is the first meta-analysis of DTI studies in individuals with ASDs. A comprehensive literature search yielded 25 ROI DTI studies that involved individuals with ASDs. Overall, DTI studies have consistently identified a reduction in anisotropy in subjects with ASDs compared with TD individuals (Tables 1 and 2). Meta-analyses of 14 studies with 330 individuals with ASDs and 313 independent TD individuals demonstrated significantly lower FA values in the CC, left UF and left SLF, and higher MD in the CC and the left and right SLF using random effects models (Figure 4).

We used a random-effects model to minimize the effects of between-study heterogeneity, and only one meta-analysis showed a severe degree of heterogeneity for FA values of the CC in the current meta-analyses (Table 3). However, previous DTI studies exhibit inherent between-study heterogeneity for other tracts and brain areas besides FA values in the CC. This heterogeneity comes from sources such as differences in data acquisition, data analysis, and subject characteristic among the studies. The current findings should thus be interpreted with caution. Indeed, the accuracy of DTI data is affected by a number of factors [75], such as the signal-to-noise ratio [76], image resolution, image distortion from magnetic susceptibility effects [77], and motion artifacts [78]. Those factors are interrelated and are influenced by the acquisition parameters including the field strength and number of directions, which differ from study to study. As the signal-to-noise ratio strongly affects diffusion tensor measures, some studies conducted several times repetitions of image acquisition to increase the signal-to-noise ratio [79]. However, the number of repetitions used varies between 1 and 15 in previous studies (Tables 1 and 2).

The current systematic review of previous literature showed that FA is the most often investigated DTI value, which ranges from 0 representing maximal isotropic diffusion of water to 1 representing maximal anisotropic diffusion. Although FA values generally reflect cellular membrane integrity, fiber myelination, fiber diameter, localized water content, and directionality [80], the abnormality underlying the reductions in FA values in subjects with ASDs is yet to be specified. As FA reductions may occur as a result of different abnormalities or a combination of abnormalities, and because there is heterogeneity in the ASD population [81], different subpopulations of people with ASDs may display FA reductions from different abnormality. We might possibly have combined FA reductions from different causes in the same meta-analysis. We expect that future study will account for the heterogeneities in people with ASDs by synthesizing an increased number of studies.

In the current study, the meta-analysis demonstrated significantly reduced FA values and increased MD values, which suggests decreased myelination or axonal densities, in the CC of ASDs compared with that of TD individuals. Decreased white matter integrity in the CC is in line with findings from a previous meta-analysis of MRI studies that focused on the CC size and demonstrated a significantly smaller than normal CC in people with ASDs [82]. The CC provides an integrated interhemispheric white matter bundle and is topographically organized [83,84]. Its disruption may result in many of the sensory, cognitive, and behavioral symptoms observed in children with ASDs [84]. In line with this notion, previous functional MRI studies [85,86] and an EEG study during a REM sleep [87] have reported interhemispheric disconnectivity, suggesting an underlying CC pathophysiology, in people with ASDs compared with TD individuals. The meta-analysis also identified similar pattern of deviations in FA of the left and MD of the bilateral SLF in ASDs. Although the SLF forms the main connection between frontal and occipital regions, this tract has extensive branching in the frontal, parietal and temporal lobes [39]. The tract has been recognized as an important structure for information exchange between Broca’s and Wernicke’s area [88,89] and may constitute underpinning of abnormal language processing [90], which is frequently observed in people with ASDs.

The current meta-analysis of FA values in the left UF demonstrated a significant reduction in people with ASDs. The importance of this intrahemispheric tract that involves the frontal lobe has also been recognized in the pathophysiology of ASDs [1]. Although there was not enough statistical power and there were no results regarding the axial diffusivity and radial diffusivity to make firm conclusions, the findings of reduced FA in combination with no change in MD may represent microstructural abnormality without gross tissue volume reduction [91]. These significant reductions in FA are concordant with the results of a previous meta-analysis of VBM studies in subjects with ASDs that reported increased white-matter volumes in the UF [92]. The UF is a bundle that connects the medial and lateral orbitofrontal cortices with the anterior portions of the temporal lobe, which are important regions for emotional and attentional processing and reward-related decision making [93-95]. The UF also includes projections into the cortical nuclei of the amygdala [96,97], a limbic region known for its involvement in human social and emotion processing [98,99] and the pathophysiology of ASDs [100-104]. Furthermore, functional MRI studies have repeatedly revealed aberrant functional connectivity between the frontal and temporal lobes in people with ASDs (for example [1]).

The current analyses demonstrated a relatively left-dominant abnormality in the association fibers in individuals with ASDs. The results of the meta-analysis may reflect leftward asymmetry in the brain, which is unrelated to the scanner, brain volume, sex and handedness in TD individuals [105-107] and ASD subjects [36,37]. Brain asymmetry is reported to be a result of typical maturation and essential for typical emotional processing, speech perception, cognition and sensory and motor functions [108-111]. The left-dominant abnormality in the current meta-analysis may be a result of atypical brain maturation in ASD individuals and a potential pathophysiology of disturbances of these functions.

Several limitations of our study should be considered. First, the limited number of included studies in the current meta-analysis did not allow us to perform a meta-regression analysis to test the effects of methodological and clinical differences among the included studies. In addition, there still remains the possibility that meta-analyses did not yield significant difference in other tracts, such as the inferior longitudinal fasciculus, because of the insufficient number of studies included. We therefore cannot emphasize the specificity of abnormalities of the CC, UF and SLF. Second, methodological differences across studies such as in the strength of the magnetic field or the number of excitations and especially regional boundaries of ROIs could have strong effects on DTI findings. In terms of regional boundaries of ROIs, the included studies differ from each other considerably. Although previous meta-analyses also integrated studies employing ROI and those utilizing tractography [33,34,112], the difference in definition of regional borders yielded considerable between-study heterogeneity. Tractography intends to cover the entire fiber, whereas the boundaries of ROIs are determined by the researchers [113]. To reduce the heterogeneity between DTI values from the entire fiber obtained by tractography and one part of fiber gained by ROI, we calculated the mean value of multiple ROIs in the case locating several ROIs within one fiber, although the attempt does not completely diminish the inherent heterogeneity. Third, the clinical characteristics of included study participants differ between studies considerably. The mean age of the participants may be one of the most important confounds because of the abnormal brain growth trajectory in the ASD brain [114-116]. Therefore, although we listed studies included in the meta-analyses in the order of the mean age of participants to enable the reader to qualitatively assess the potential effects of age on effect size, the current meta-analysis could not exclude the possibility that age had effects on FA levels in brain areas such as the CC, UF and SLF. Fourth, although previous studies have reported abnormalities in projection fibers [73] and fibers that connect cerebrum and cerebellum [67,68], and suggested that they potentially underlie the pathophysiology of abnormal behavior in individuals with ASD, we could not conduct meta-analyses because of a lack of sufficient studies. Lastly, as we have reviewed, there are accumulative studies that involved voxel-based analysis to investigate tract abnormality in individuals with ASDs. Although we could not conduct a meta-analysis of these studies due to their heterogeneities, in terms of the differences between TBSS and ordinary voxel-wise analysis and small volume correction, there is no doubt that these studies and their meta-analysis could contribute in the future.

## Conclusions

DTI studies involving individuals with ASDs demonstrated significantly low FA values in the CC and left UF and high MD in the CC and SLF bilaterally compared with TD individuals. Although some confounds could not be controlled for because of the limited number of included studies, the current meta-analysis strongly demonstrates that changes in white matter integrity in ASDs are localized in the CC, UF and SLF among association and commissural fibers. The current findings further support the long-distance underconnectivity hypothesis of ASDs.

## Abbreviations

ASD: Autism spectrum disorder; CC: Corpus callosum; DTI: Diffusion tensor imaging; FA: Fractional anisotropy; MD: Mean diffusivity; MRI: Magnetic resonance imaging; ROI: Region of interest; SLF: Superior longitudinal fasciculus; TBSS: Tract-based spatial statistics; TD: Typically developing; UF: Uncinate fasciculus; WBA: Whole brain analysis.

## Competing interests

The authors declare that they have no conflicts of interest.

## Authors’ contributions

YA and YN performed the study screenings independently. YA performed all of the data extraction and calculated the effect sizes twice to avoid careless mistakes. YN further performed the data extraction and an independent calculation of the effect sizes. YA, OA and HY wrote the paper. All authors read and approved the final manuscript.

## Supplementary Material

Additional file 1 **a figure showing the one-study-removed sensitivity analysis of the corpus callosum (CC) and left uncinate fasciculus (UF).** One-study-removed sensitivity analysis of fractional anisotropy (FA) (upper) and mean diffusivity (MD) (middle) in the CC and MD in the left UF (lower). Mean and standard deviation of age of individuals with ASDs are demonstrated in the end of each study name. Studies are lined in the order of mean age from the youngest (top) to the oldest (bottom). Click here for file

Additional file 2 **a figure showing the one-study-removed sensitivity analysis of the superior longitudinal fasciculus.** One-study-removed sensitivity analysis of fractional anisotropy (FA) (upper) and mean diffusivity (MD) (middle) in the left superior longitudinal fasciculus (SLF) and MD in the right SLF (lower). Mean and standard deviation of age of individuals with ASDs are demonstrated in the end of each study name. Studies are lined in the order of mean age from the youngest (top) to the oldest (bottom). Click here for file

Additional file 3 **a figure showing funnel plots of meta-analyses.** Funnel plots of the meta-analyses of fractional anisotropy (FA) in the corpus callosum (CC) (a), mean diffusivity (MD) in the CC (b), FA in the left (lt) cingulum (Cg) (c), FA in the right (rt) Cg (d), FA in the lt uncinate fasciculus (UF) (e), MD in the lt UF (f), FA in the rt UF (g), MD in the rt UF (h), FA in the lt superior longitudinal fasciculus (SLF) (i), FA in the rt SLF (j), MD in the lt SLF (k), MD in the rt SLF (l), FA in the lt inferior longitudinal fasciculus (ILF) (m), FA in the rt ILF (n), FA in the lt inferior frontal occipital fasciculus (IFOF) (o), and FA in the rt IFOF (p). Click here for file
