# Supplementary figures and images for: Comparison of white matter integrity between autism spectrum disorder subjects and typically developing individuals: a meta-analysis of diffusion tensor imaging tractography studies
Source: Mol Autism. 2013 Jul 22;4:25. doi: 10.1186/2040-2392-4-25 (PMC3726469; doi:10.1186/2040-2392-4-25)

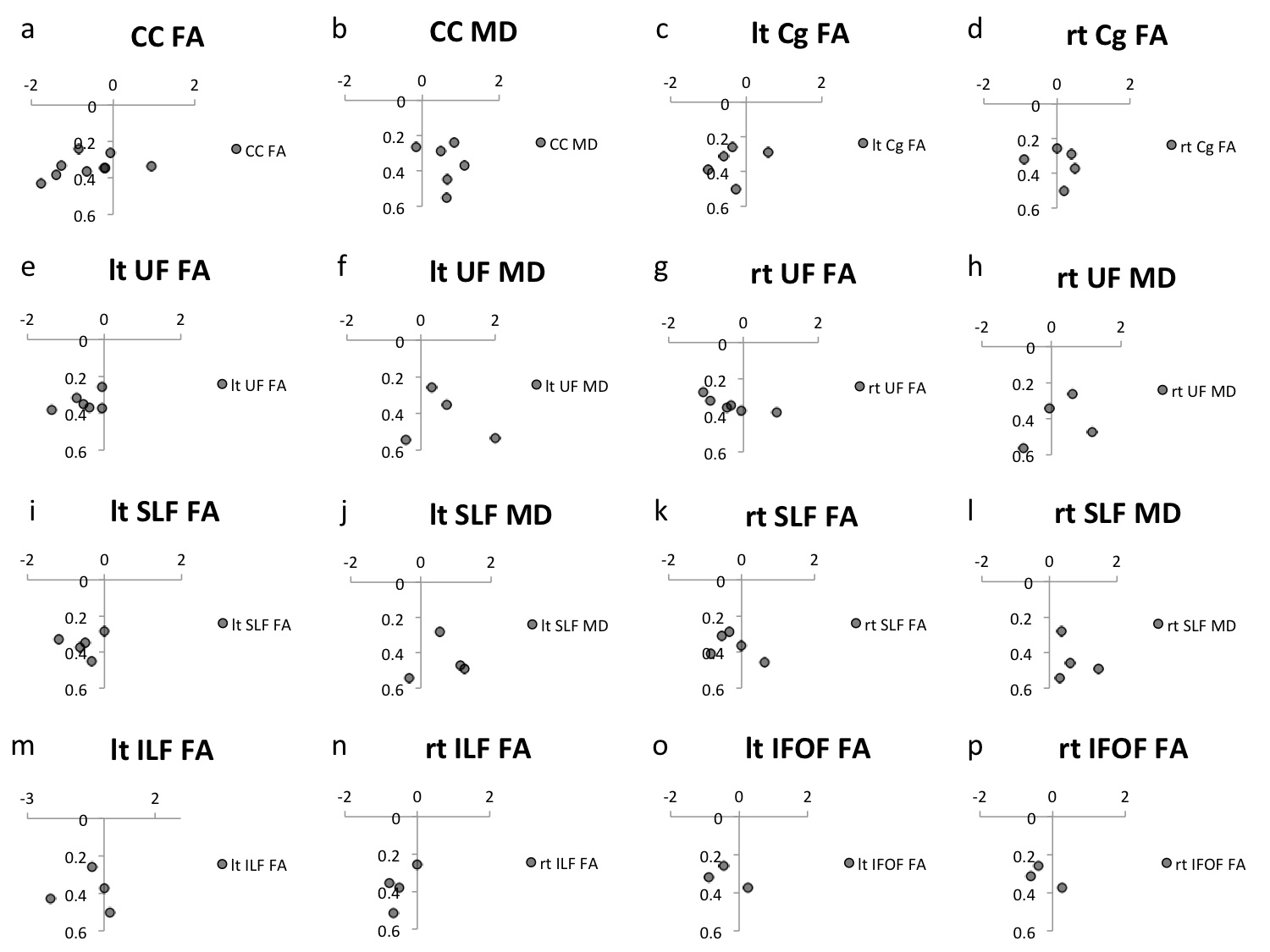

Supplement: Additional file 1 — a figure showing the one-study-removed sensitivity analysis of the corpus callosum (CC) and left uncinate fasciculus (UF). One-study-removed sensitivity analysis of fractional anisotropy (FA) (upper) and mean diffusivity (MD) (middle) in the CC and MD in the left UF (lower). Mean and standard deviation of age of individuals with ASDs are demonstrated in the end of each study name. Studies are lined in the order of mean age from the youngest (top) to the oldest (bottom). [file 2040-2392-4-25-S1.jpeg]

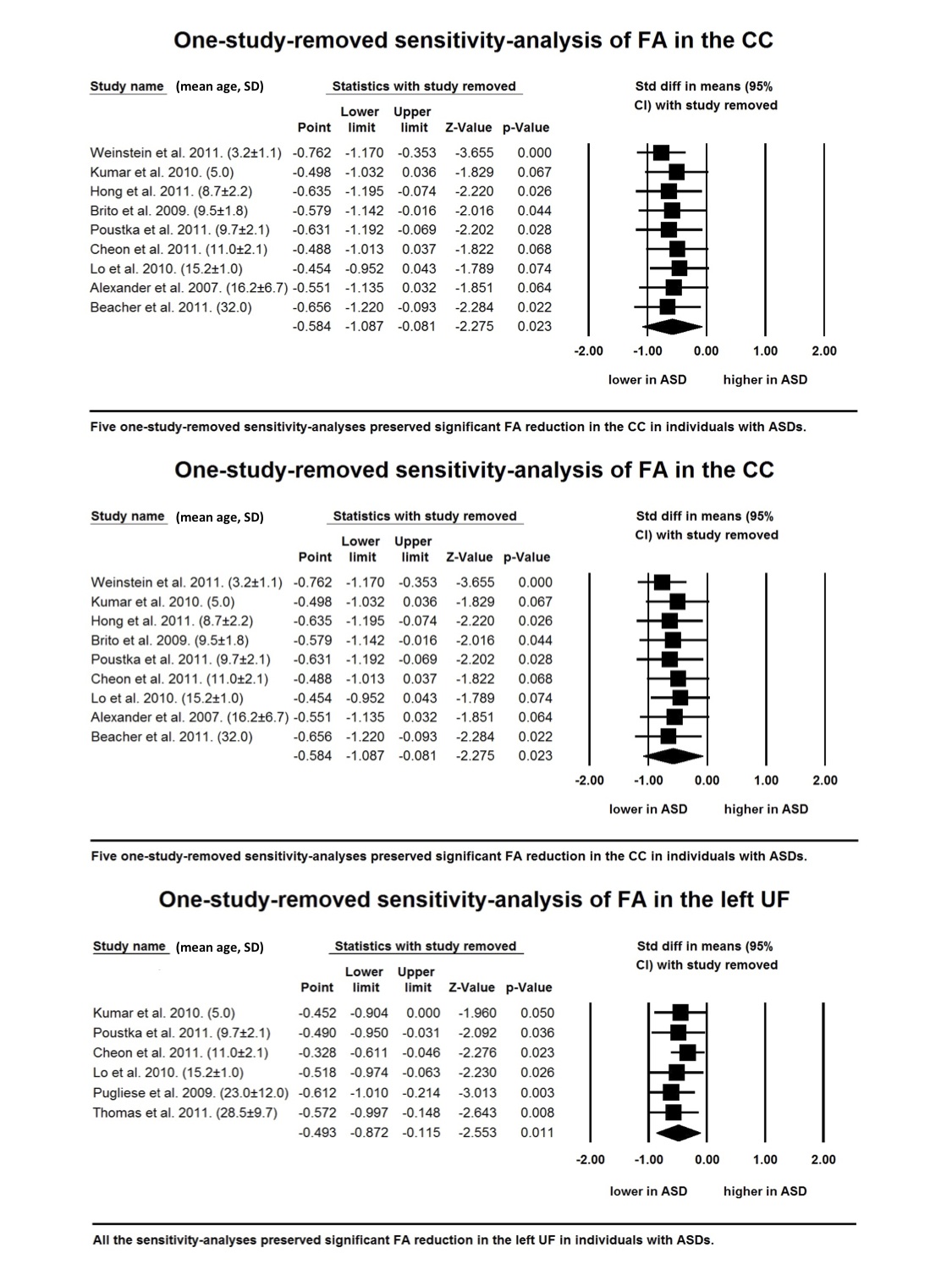

Supplement: Additional file 2 — a figure showing the one-study-removed sensitivity analysis of the superior longitudinal fasciculus. One-study-removed sensitivity analysis of fractional anisotropy (FA) (upper) and mean diffusivity (MD) (middle) in the left superior longitudinal fasciculus (SLF) and MD in the right SLF (lower). Mean and standard deviation of age of individuals with ASDs are demonstrated in the end of each study name. Studies are lined in the order of mean age from the youngest (top) to the oldest (bottom). [file 2040-2392-4-25-S2.jpeg]

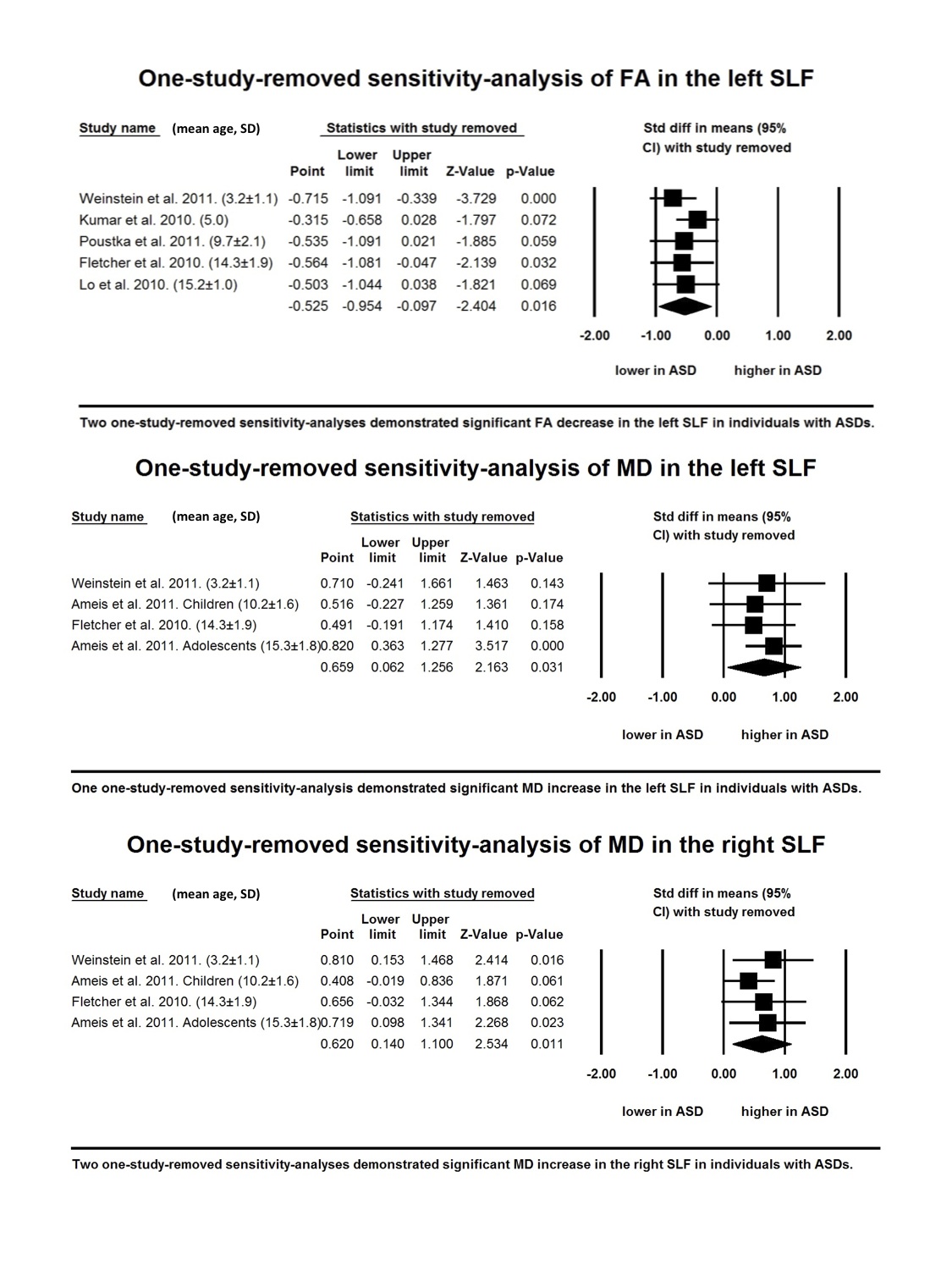

Supplement: Additional file 3 — a figure showing funnel plots of meta-analyses. Funnel plots of the meta-analyses of fractional anisotropy (FA) in the corpus callosum (CC) (a), mean diffusivity (MD) in the CC (b), FA in the left (lt) cingulum (Cg) (c), FA in the right (rt) Cg (d), FA in the lt uncinate fasciculus (UF) (e), MD in the lt UF (f), FA in the rt UF (g), MD in the rt UF (h), FA in the lt superior longitudinal fasciculus (SLF) (i), FA in the rt SLF (j), MD in the lt SLF (k), MD in the rt SLF (l), FA in the lt inferior longitudinal fasciculus (ILF) (m), FA in the rt ILF (n), FA in the lt inferior frontal occipital fasciculus (IFOF) (o), and FA in the rt IFOF (p). [file 2040-2392-4-25-S3.jpeg]
